# Mesoporous nitrogen-doped TiO_2 _sphere applied for quasi-solid-state dye-sensitized solar cell

**DOI:** 10.1186/1556-276X-6-606

**Published:** 2011-11-24

**Authors:** Peng Xiang, Xiong Li, Heng Wang, Guanghui Liu, Ting Shu, Ziming Zhou, Zhiliang Ku, Yaoguang Rong, Mi Xu, Linfeng Liu, Min Hu, Ying Yang, Wei Chen, Tongfa Liu, Meili Zhang, Hongwei Han

**Affiliations:** 1 Michael Grätzel Center for Mesoscopic Solar Cells, Wuhan National Laboratory for Optoelectronics, Huazhong University of Science and Technology, Wuhan, 430074, Hubei, People's Republic of China

## Abstract

A mesoscopic nitrogen-doped TiO_2 _sphere has been developed for a quasi-solid-state dye-sensitized solar cell [DSSC]. Compared with the undoped TiO_2 _sphere, the quasi-solid-state DSSC based on the nitrogen-doped TiO_2 _sphere shows more excellent photovoltaic performance. The photoelectrochemistry of electrodes based on nitrogen-doped and undoped TiO_2 _spheres was characterized with Mott-Schottky analysis, intensity modulated photocurrent spectroscopy, and electrochemical impedance spectroscopy, which indicated that both the quasi-Fermi level and the charge transport of the photoelectrode were improved after being doped with nitrogen. As a result, a photoelectric conversion efficiency of 6.01% was obtained for the quasi-solid-state DSSC.

## Introduction

Since a mesoscopic TiO_2_-based dye-sensitized solar cell [DSSC] was reported by O' Regan and Grätzel in 1991 [1], DSSC has emerged as a promising candidate for the practical photovoltaic application due to its low manufacturing cost and high energy conversion efficiency. Even though DSSC is now commercially available, market expansion is still limited because of the use of a liquid electrolyte, which always signifies instability due to the leakage of the solvent and the erosion of the electrode. It seems a good way to replace the liquid electrolyte by a solid-state or quasi-solid-state medium [2,3]. Unfortunately, compared with liquid electrolyte, solid-state or quasi-solid-state DSSC still presents lower energy conversion efficiency. Therefore, how to improve the performance of DSSC composing of solid-state or quasi-solid-state medium is still a big issue.

As one of the key components, mesoscopic TiO_2 _film plays an important role in determining the performance of DSSC, which assumes both the task of dye anchorage and charge carrier transport. Over the past decade, substantial efforts have been made to improve the performance of DSSC through the reformation of the TiO_2 _film. The first strategy is to increase the light absorption efficiency of the photoanode by increasing the surface area of the TiO_2 _film, which provides sufficient room for dye loading. Another strategy is to improve the electron injection efficiency by adjusting the conduction band edge to match the LUMO of the dye [4]. The last strategy is to increase the charge collection efficiency through the improvement of the electron transport or lifetime [5].

For an effective DSSC, the key parameters of the mesoscopic TiO_2 _film such as porosity, pore size distribution, light scattering, electron percolation, and conduction band edge should be coordinated to the characterization of the dye and electrolyte medium, which could be controlled by precursor chemistry, hydrothermal growth temperature, binder addition, doping materials, and sintering conditions [6]. Recently, Chen et al. [7] reported a mesoporous anatase TiO_2 _bead with high surface area and controllable pore size for DSSC, which indicated that the DSSC employing mesoporous TiO_2 _sphere has demonstrated longer electron diffusion lengths and extended electron lifetimes over Degussa P25 titania electrodes due to the favorable interconnected, densely packed nanocrystalline TiO_2 _particles inside the spheres, resulting in the improvement of the parameters. In 2005, Ma et al. [8] reported nitrogen-doped TiO_2 _particles for DSSC that enhanced the incident photon-to-current conversion efficiency within the spectrum of 380 to 520 nm and 550 to 750 nm; as a result, the short-circuit photocurrent density was pronouncedly improved. After that, Tian et al. [9] found that the position of the flatband potential edge was shifted to negative when doping nitrogen into the TiO_2 _film, which is attributed to the formation of an O-Ti-N bond and results in the improvement of the open-circuit voltage in DSSC.

Therefore, it could be expected to promote the performance of DSSC from the improvement of both the short-circuit photocurrent density and the open-circuit voltage with the nitrogen-doped TiO_2 _sphere. Herein, we attempted to synthesize a crystalline nitrogen-doped TiO_2 _sphere under the hydrothermal condition and apply it in quasi-solid-state DSSC. The results indicated that with the nitrogen-doped TiO_2 _sphere, the parameters of quasi-solid-state DSSC were improved in both the short-circuit photocurrent density and the open-circuit voltage. As a result, a power conversion efficiency up to 6.01% of quasi-solid-state DSSC was obtained under air mass [AM] 1.5 sunlight at 100 mW/cm^2^.

## Experimental details

Mesoporous TiO_2 _spheres were synthesized with the hydrothermal method as follows: 8 g dodecylamine and 8 g titanium isopropoxide [TIP] were mixed with 360 mL ethanol. The urea solution was added into the dodecylamine-TIP mixture solution under vigorous stirring at ambient temperature. The molar ratio of the urea to TIP was adjusted to 0, 4, 8, and 16. Twelve hours later, the white TiO_2 _suspension was transferred into a Teflon-lined autoclave and then heated at 210°C. After 12 h, the nitrogen-doped TiO_2 _spheres were obtained and marked as T_N0_, T_N1_, T_N2_, and T_N3_, respectively.

Mesoscopic nitrogen-doped or undoped TiO_2 _sphere pastes for screen print were prepared as reported [10]. Hydroxypropyl cellulose was dissolved in ethanol to form 10 wt.% ethanolic mixture. Two grams of mesoscopic nitrogen-doped TiO_2 _sphere, 5 g 10 wt.% ethanolic mixture, 8.1 g terpineol, and 10 mL ethanol were mixed by ball milling for 12 h. Then, the slurry was sonicated for 30 min and concentrated to a paste with 20 wt.% TiO_2_.

The mesoscopic nitrogen-doped or undoped TiO_2 _sphere-based photoelectrode was coated on a fluorine-doped tin oxide [FTO] glass (TEC15; Pilkington TEC Glass™, IL, USA) by using a doctor blade and then sintered at 450°C for 30 min. The thickness of nitrogen-doped or undoped TiO_2 _sphere film was controlled by the thickness of scotch tape. The photoelectrode with a 2-μm TiO_2 _sphere layer was used for Mott-Schottky [MS] analysis. The electrode with a 12-μm TiO_2 _sphere film was dyed with N719 ethanolic solution. A sandwich structure quasi-solid-state DSSC containing the mesoscopic TiO_2 _sphere-based electrode, platinized counter electrode, and quasi-solid-state electrolyte was performed for photocurrent-voltage testing, which was sandwiched with a 100-μm film of Surlyn (DuPont Surlyn, Wilmington, DE, USA) and then heated at 100°C for sealing.

The surface appearance of their films on FTO glass was performed using a Sirion 200 field emission scanning electron microscopy [FE-SEM] (FEI Company, Eindhoven, the Netherlands). Veeco Dektak 150 (Veeco Instruments Inc., NY, USA) was used to detect the thickness of the TiO_2 _film. MS analysis was performed in a three-electrode cell under a dark condition, where the T_N0_- and T_N2_-based electrodes without dye (ca. 2 μm) were used as the work electrode, a saturated calomel electrode [SCE] served as the reference electrode, a platinum wire was used as the counter electrode, and the active area was 0.36 cm^2^. The ethylene carbonate and propylene carbonate (1:1 volume ratio) solution containing 0.01 M iodine [I_2_] and 0.1 M potassium iodide was used as an electrolyte for MS analysis. Intensity modulated photocurrent spectroscopy [IMPS] was characterized within T_N0_- and T_N2_-based DSSC and performed with a green light emitting diode (*λ*_max _= 535 nm) driven by Zahner Zenium. The photocurrent density-voltage curves of the quasi-solid-state DSSC were perfomed with a Keithley 2400 SourceMeter (Keithley Instruments, Inc., Ohio, USA) under illumination with an Oriel solar simulator (Newport Corporation, CA, USA) composed of a 1, 000-W xenon arc lamp and AM 1.5 G filters. Light intensity was calibrated with a normative silicon cell. The electrochemical impedance spectroscopy [EIS] of the quasi-solid-state DSSC was characterized by a potentiostat (M2273; EG&G Inc., MD, USA) with a frequency range from 100 mHz to 1 MHz.

## Results and discussion

**Figure 1:**
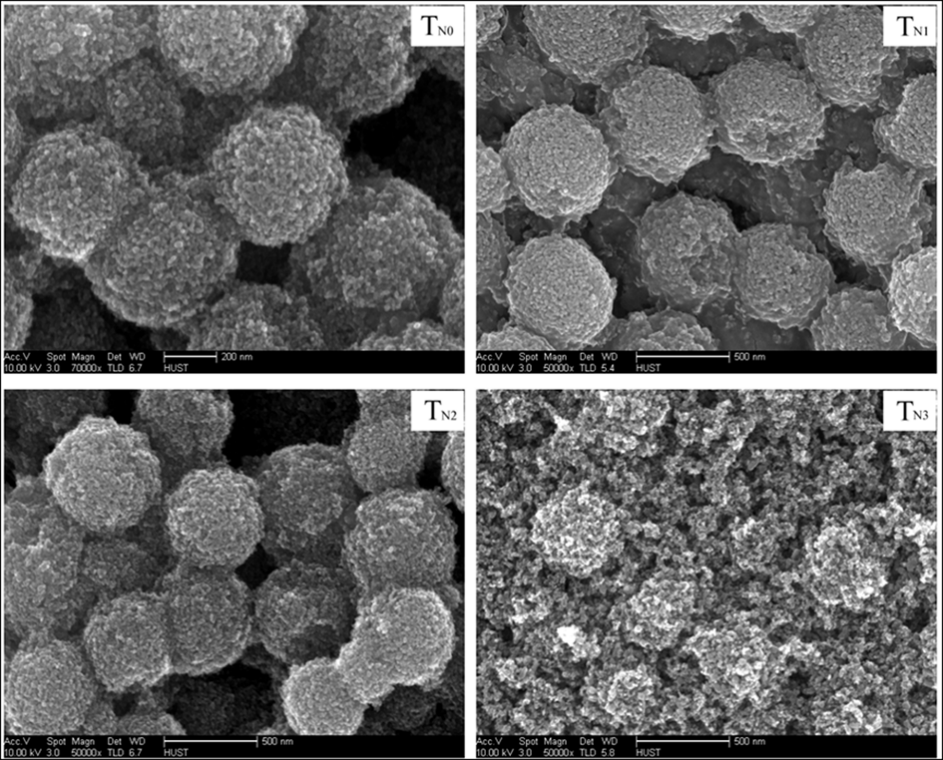
**SEM images of nitrogen-undoped and doped TiO_2 _spheres based films**.

Figure 1 shows the SEM images of the TiO_2 _films using T_N0_, T_N1_, T_N2_, and T_N3 _powders. It could be found that films using T_N0_, T_N1_, and T_N2 _are composed of the interconnected mesoscopic spheres with the size around 550 nm, but the film using T_N3 _is composed of a three-dimensional network of interconnected nanoparticles. The TiO_2 _film composing of large mesoscopic spheres with considerable quantities and small nanocrystalline particles presents the ability to scatter light and also provides sufficient surface area for the dye loading. However, urea and dodecylamine are used as the nitrogen source and dispersant, forming mesoporous TiO_2 _spheres during the thermal treatment, respectively. Too much urea could destroy the accompaniment of the TiO_2 _sphere. Therefore, the mesoporous spheres are dispersed into small particles with the increase of urea content in the precursor solution as shown in the film using T_N3_.

**Figure 2:**
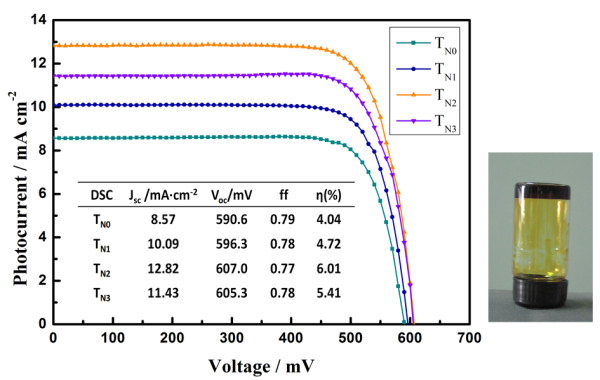
**Current-voltage curves of quasi-solid-state DSSCs with nitrogen-doped and undoped TiO_2 _sphere-based electrodes**.

Figure 2 presents the photocurrent density-voltage curves of the DSSC using T_N0_, T_N1_, T_N2_, and T_N3 _as photoelectrodes. Here the electrolyte is 10 wt.% Poly(ethylene oxide) (*M*_w _= 2 × 10^6 ^g·mol^-1^), 0.1 M I_2_, 0.1 M lithium iodide, 0.6 M 1, 2-dimethyl-3-propyl imidazolium iodide, and 0.45 M *N*-methyl-benzimidazole 3-methoxypropionitrile solution [11]. An inverted vessel containing this electrolyte is shown in the inserted picture of Figure 2 and exhibits the characterization of the quasi-solid-state DSSC. The parameters of DSSCs are summarized in the inserted table, which demonstrates that compared with the undoped-nitrogen TiO_2 _sphere-based quasi-solid-state DSSC, all the performance of doped-nitrogen TiO_2 _sphere-based quasi-solid-state DSSC was improved. With the increase of nitrogen content within the TiO_2 _spheres, the short-circuit photocurrent density [*J*_sc_] is increased from 8.57 mA·cm^-2 ^of the undoped TiO_2 _sphere-based quasi-solid-state DSSC (T_N0_) to 10.09 mA·cm^-2 ^of the T_N1 _and then reached to a maximum value of 12.82 mA·cm^-2 ^of the T_N2_. However, when the nitrogen content within the sphere is increased further, the *J*_sc _is decreased to 11.43 mA·cm^-2 ^of the T_N3_. Compared with *J*_sc_, the open-circuit voltage [*V*_oc_] is increased within a small amplitude with the increase of nitrogen content within the TiO_2 _spheres, which exhibits 590.6 mV for T_N0_, 596.3 mV for T_N1_, 607.0 mV for T_N2_, and 605.3 mV for T_N3_, and the fill factor is almost at a constant value of around 0.78, which is presented as follows: 0.79 for T_N0_, 0.78 for T_N1_, 0.77 for T_N2_, and 0.78 for T_N3_. As a result, the highest energy conversion efficiency [*η*] of 6.01% was obtained for T_N2_-based quasi-solid-state DSSC, which is 49% higher than that of T_N0 _(*η *= 4.04%), 27% higher than that of T_N1 _(*η *= 4.72%), and 11% higher than that of the T_N3_-based device (*η *= 5.41%).

**Figure 3:**
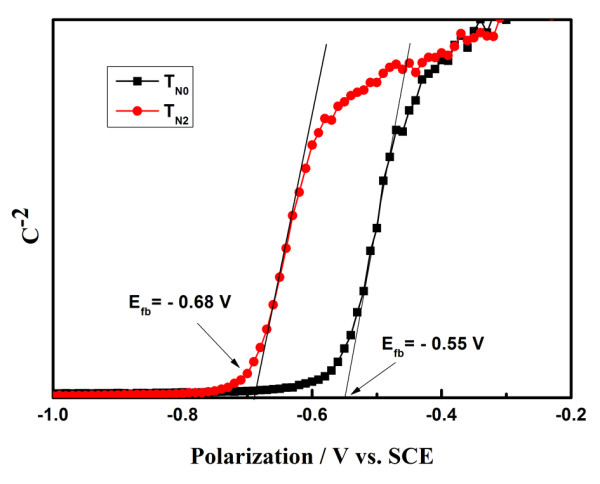
**Mott-Schottky plots of an electrode using T_N0 _and T_N2_**.

In order to ascertain the role of doping nitrogen, two typical mesoscopic TiO_2 _sphere photoeletrodes with (T_N2_) and without doping nitrogen (T_N0_) were investigated by MS analysis, EIS, and IMPS. Normally, the *V*_oc _of DSSC could be improved by retarding interfacial recombination, shifting the conduction band of TiO_2 _to more negative potentials, and engineering a more favorable equilibrium Fermi-level position [12]. The flat band potential [*E*_fb_] is a very useful quantity in photoelectrochemistry as it facilitates location of the energetic position of the valence and conduction band edge of a given semiconductor material [13], which could be detected by MS analysis [9-12]. Figure 3 presents the MS plots of T_N0_- and T_N2_-based electrodes. E_fb _could be calculated by the following equation [14].

$${\left(C_{\text{sc}}\right)}^{-\text{2}}=\text{ 2 }\left(E-E_{\text{fb}}-kT∕e\right)\;∕N_{\text{D}}\varepsilon \varepsilon_{0}eA^{\text{2}}$$

where *C*_sc _is the space charge capacity, *N*_D _is the electron density, *ε *is the dielectric constant, *ε*_0 _is the permittivity of vacuum, and *A *is the active surface area. *E*_fb _is the intercept extrapolating a straight line from the plot of the square of the space charge capacity against the applied voltage, which found that the *E*_fb _of the electrode using T_N0 _and T_N2 _are about -0.55 V and -0.68 V (vs. SCE), respectively. Compared with the undoped TiO_2 _sphere-based electrode, the *E*_fb _of the nitrogen-doped TiO_2 _sphere-based electrode is shifted to about 0.13 V negatively, which suggests that a higher quasi-Fermi level could be obtained by doping nitrogen, resulting in the enhancement of *V*_oc _of the mesoscopic nitrogen-doped TiO_2 _sphere-based DSSC.

**Figure 4:**
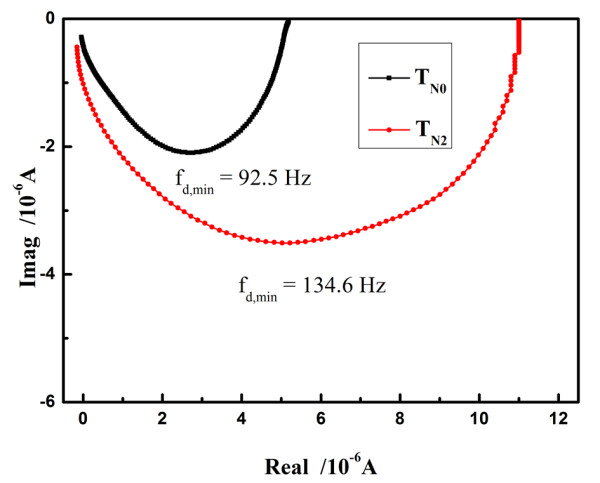
**IMPS plots of T_N0_- and T_N2_-based quasi-solid-state DSSCs**.

The charge transport within the nanocrystalline semiconductor of the photoelectrode could be detected by IMPS, and Figure 4 presents the IMPS of the photoelectrode using T_N0 _and T_N2_. The transport time [*τ*_d_] of the injected electrons through the TiO_2 _film can be calculated from the equation *τ*_d _= 1/(2π*f*_d, min_) [15], where *f*_d, min _is the characteristic frequency at the minimum of the IMPS imaginary component. The simulation results show that *τ*_d _values are estimated to be 1.72 and 1.18 ms in the films using T_N0 _and T_N2_, respectively. In addition, the electron diffusion coefficient [*D*_n_] could be calculated from the equation *D*_n _= *d*^2^/(2.35*τ*_d_) [15], where *d *is the thickness of the photoeletrode. Therefore, the *D*_n _in the films using T_N0 _and T_N2 _are calculated to be 9.9 × 10^-6 ^and 1.44 × 10^-5 ^cm^2^·s^-1^, respectively. It is clear that the charge transport within the T_N2 _film is faster than that of the T_N0 _film, which suggests that the charge collection efficiency and *J*_sc _of DSSC could be improved by nitrogen doping.

**Figure 5:**
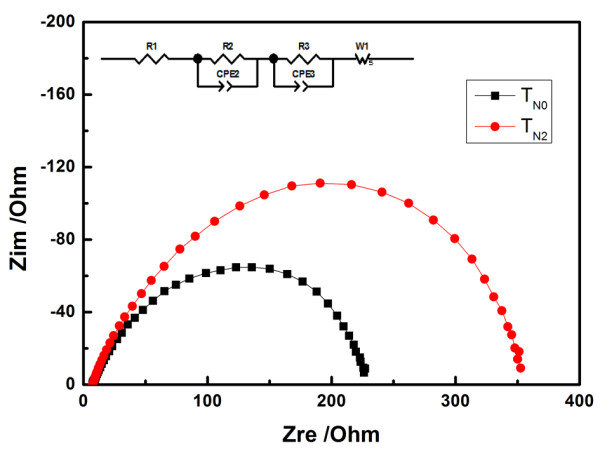
**Nyquist plots of T_N0_- and T_N2_-based quasi-solid-state DSSCs**.

EIS was carred out in the dark condition to investigate the discrepancy of the interfacial recombination between the electrodes using T_N0 _and T_N2_. Figure 5 presents the typical Nyquist plots of the T_N0_- and T_N2_-based cells. The interfacial impedance related to the electron transfer from the conduction band of TiO_2 _to the electrolyte can be described by the semicircle of the middle frequency region in the Nyquist plot. Their charge transfer resistance [*R*_ct_] values are evaluated by fitting the spectra with the equivalent circuit shown in the inset, which shows 281 Ω of T_N0_- and 362 Ω of T_N2_-based cells. Compared with the pure TiO_2 _sphere-based cell, the *R*_ct _of the mesoscopic nitrogen-doped TiO_2 _sphere-based cell is increased to 29%, suggesting that the interface resistance between the TiO_2 _sphere electrode and electrolyte is increased by introducing nitrogen. Therefore, the interface recombination between photoelectrode and electrolyte within DSSC would be retarded with this nitrogen-doped TiO_2 _sphere, resulting in the decrease of dark current and the improvement of *J*_sc _and *V*_oc_.

## Conclusions

A series of mesoscopic nitrogen-doped and undoped TiO_2 _spheres for quasi-solid-state DSSC have been prepared by the hydrothermal method. The effects of nitrogen within TiO_2 _spheres on the photovoltaic performance have been investigated, which show that the introduction of nitrogen increases *J*_sc_, *V*_oc_, and efficiency. The photoelectrochemistry of nitrogen-doped and undoped TiO_2 _sphere-based electrodes was performed with MS, IMPS, and EIS. The results indicated that the quasi-Fermi level and the charge transport of the photoelectrode were improved, and the interfacial recombination was retarded after being doped with nitrogen within a mesoscopic sphere-based electrode. As a result, a DSSC with a power conversion efficiency of 6.01% under AM 1.5 sunlight at 100 mW/cm^2 ^has been obtained with N719 dye in combination with a quasi-solid state electrolyte. Higher parameter DSSC could be expected with this mesoscopic nitrogen-doped TiO_2 _sphere.

## Competing interests

The authors declare that they have no competing interests.

## Authors' contributions

PX carried out the DSSC studies, participated in the sequence alignment and drafted the manuscript. XL, HW, GL, TS, ZZ, and WC participated in the design of the study and performed the statistical analysis. ZK, YR, MX, LL, MH, YY, TL, and MZ carried out material synthesis. HH conceived of the study and participated in its design and coordination. All authors read and approved the final manuscript.
